# Supplementary material for: Are carbon emissions trading and green financial instruments synergistic? -Comprehensive quantitative research based on content analysis
Source: PLoS One. 2024 Mar 7;19(3):e0298601. doi: 10.1371/journal.pone.0298601 (PMC10919601; doi:10.1371/journal.pone.0298601)
Supplement: S1 Data — (ZIP) [file pone.0298601.s001.zip › Data/地级市Z_F工作报告.zip!/╕ú╜¿/╧├├┼╩╨/└┤╫╘╣1⁄2╓┌║┼í╛┬φ┐╦╩2╛▌═°í┐_2009.docx]

各位代表：

　　现在，我代表市人民政府向大会作政府工作报告，请予审议，并请市政协委员和各位列席人员提出意见。

　　一、2008年的工作回顾

　　2008年，是改革开放三十周年。我们在中共厦门市委的领导下，高举中国特色社会主义伟大旗帜，以邓小平理论和“三个代表”重要思想为指导，深入贯彻落实科学发展观，努力构建社会主义和谐社会，全面融入海峡西岸经济区建设大局，持续推进新一轮跨越式发展，积极有效应对国际金融危机带来的严重冲击，保持了我市经济社会健康平稳较快发展，基本实现了发展速度、质量、效益相统一。初步统计，全年地区生产总值1560亿元，增长11.1%；规模以上工业总产值2929.6亿元，增长10%；全社会固定资产投资928.3亿元，与上年度基本持平；外贸进出口总值453.9亿美元，增长14.1%，其中，出口293.9亿美元，增长15%，进口159.9亿美元，增长12.4%；实际利用外资20.4亿美元，增长60.6%；财政总收入410.1亿元，增长17.7%，其中地方级财政收入220.2亿元，增长18.1%；城镇居民人均可支配收入23948元，增长11.4%，农民人均纯收入8475元，增长11%；社会消费品零售总额418.9亿元，增长15.7%；居民消费价格指数104.9，城镇登记失业率4.14%，人口自然增长率8.2‰。二氧化硫排放量削减20.34%，化学需氧量削减4.88%，单位生产总值能耗降低2.52%的目标预计可以完成。

　　一年来，我们主要做了以下工作：

　　（一）正确研判经济形势，积极应对国际金融危机

　　面对严峻复杂的国际国内经济形势，我们始终保持清醒，正视风险，坚定信心，积极应对，有效地减缓了国际金融危机带来的冲击。一是及时发出预警，提早研究对策措施。举办专题知识讲座，邀请专家就美国次贷危机及其对厦门经济的影响进行分析，要求各部门各单位高度关注，深入调研。召开工业、金融、房地产、出口、投资等专题座谈会，寻找应对措施，研究解决问题。二是及时出台扶持政策，做好跟踪服务。出台促进外贸企业出口、支持中小企业和民营经济发展、促进房地产业健康发展、促进扩大内需等方面的具体政策措施，着力解决经济运行中的突出矛盾和关键问题，进一步减轻企业负担。市政府与中国信保签订战略合作协议，扩大企业出口信用保险规模，提升企业规避风险和融资的能力。加快企业出口退税速度，及时通过各种渠道向企业宣传国家、省、市出台的扶持政策，帮助企业用好用足政策。三是千方百计保障项目资金需求。加强政、银、企良性互动，积极协调解决企业融资困难，想方设法帮助企业渡过难关。确定从2008年第四季度到2010年集中投入财政性资金380亿元，优先实施一批生产性项目、重大基础设施项目和社会事业项目，带动全社会投资2000亿元，力争尽快建成，尽快发挥经济和社会效益。积极争取国家增投支持，已有一批项目列入中央新增投资计划。

　　（二）着力转变经济发展方式，产业结构进一步优化

　　坚持优先发展工业，以工业带动第三产业，提升第一产业。加快构建海峡西岸强大的先进制造业基地，工业支撑作用进一步增强，高新技术产业、新兴产业和特色产业发展加快，第三产业比重提高0.6个百分点，产业素质进一步提升。

　　大力发展先进制造业。通士达节能光源制造基地、金桥卷烟生产线技改等项目加快推进，安台创新科技、银鹭重工等企业竣工投产。达运精密、厦船重工、古龙罐头等一批优势企业逆势增长，友达光电、太古飞机维修、戴尔计算机等7家企业净增产值均超10亿元，正新实业、林德叉车、松霖科技等78家企业净增产值均超亿元，液晶显示屏、节能灯、客车、工程机械等地产品国际市场进一步拓展。工业产品产销率达到99.93%，比上年提高1.07个百分点。新增3个中国驰名商标，高新技术企业产值占全市工业总产值比重达58.6%。

　　着力提升自主创新能力。电力电器产业基地成为国家级特色产业基地，中科院城市环境研究所一期投入使用，厦门IC设计公共服务平台、国家（厦门）机动车污染控制中心等重大科技平台建设进展顺利，国家场（厂）内机动车辆、半导体发光器件应用产品质检中心建设稳步推进。新增厦门大学醇醚酯化工清洁生产国家工程实验室，宏发电声、三安电子国家级企业技术中心，金龙联合汽车、特宝生物、厦门国际银行博士后工作站，厦门钨业成为首批国家级创新型企业，厦门金鹭获国家科技进步二等奖，国家一类新药重组戊肝疫苗进入Ⅲ期临床试验，全市发明专利授权量增长75.9%。

　　加快发展现代服务业。海沧保税港区获批并加快建设，象屿保税区、保税物流园区和东渡港区三区整合取得实效。开通赣州—厦门集装箱海铁联运定期班列和东北—厦门—赣州海铁联运航线，顺利推进赣州、三明等内陆“无水港”项目。中海、中远等国内大型航运商在厦投入大型内贸集装箱运输船舶，内贸集装箱增长29.7%。马士基、以星航运等国际航运商加大在厦国际中转业务，港口集装箱国际中转业务增长2.4倍。厦门港货物年吞吐量9702万吨，集装箱年吞吐量突破500万标箱。出台扶持企业上市政策，合兴包装、安妮股份等4家企业成功上市。观音山等商务营运中心区引进了一批企业的地区总部、销售中心、结算中心和研发中心。实现了大陆游客从厦门口岸乘坐邮轮出境旅游，全年共接待邮轮游客近6.7万人次，为建设厦门国际邮轮母港奠定良好基础。游艇（帆船）产业基地落户厦门，观音山滨海旅游休闲区、奥林匹克博物馆等一批新景点顺利建成，磐基假日、牡丹等高星级酒店建成营业。会展中心二期和国际会议中心投入使用，成功举办第十二届中国国际投资贸易洽谈会、首届国际动漫节、厦门国际海洋周、石材展等重大会展，全年会展超过1700场次。接待境内外旅游者2194.1万人次，其中境外游客125.6万人次。引进一批动漫网游企业，国家动画产业基地、国内首个国家软件与集成电路人才国际培训基地落户厦门，软件业产值增长30%。厦门影视作品首次在央视少儿频道黄金时段播出。图书印刷、商品油画、钢琴制造及琴行培训业、艺术品制造及拍卖业和艺术表演等文化创意产业加快发展。

　　推进节能减排和资源综合利用。认真落实《厦门市节约能源条例》，制定实施《固定资产投资项目节能评估和审查办法》，强化节能减排目标责任制，制定节能技术和产品推荐目录，扶持节能示范工程和节能技改项目，大力推广节能技术和产品。调整优化用地结构，提高土地利用效率。完成污染源普查，主动淘汰落后产能，主要污染物减排工作走在全国重点城市前列。开展循环经济试点，推进海水综合利用、雨水利用和中水回用，节水型城市创建通过国家验收。

　　（三）强化城市建设管理，中心城市综合承载能力提升

　　扎实推进环东海域等重大片区及一大批事关长远的交通、港口、市政、环保等重大基础设施的规划和建设，全年完成重点项目投资382亿元。快速公交系统（BRT）一期、集美大桥、杏林大桥、疏港路高架二期及成功大道、环岛干道部分路段建成通车，天马山隧道、龙门岭隧道全线贯通。福厦、厦深、龙厦高速铁路等一批重大基础设施项目加快推进，环东海域、五缘湾、杏林湾、湖边水库、厦门（新）站等重大片区项目建设进展顺利，厦漳跨海大桥、沈海高速公路扩建（厦门段）等项目开工建设。新增万吨级以上泊位3个。东部供水工程、220千伏围里输变电等一批重大项目建成投入使用。数字化城管系统一期工程投入试运行。成为全国首个采用TD－SCDMA技术标准建设的“无线城市”。

　　加强城市生态环境建设，加快推进液化天然气（LNG）等项目建设，扩建改建污水处理厂和垃圾处理工程，基本建成后坑垃圾焚烧发电厂、东部固废中心垃圾填埋场等一批市政工程，推广应用水煤浆等新技术和绿色照明，完成一批新建道路、桥梁的景观美化亮化，建成第五期LED夜景工程。加大海域清淤和综合整治力度，开展筼筜湖污染源专项治理，基本完成环东海域第一阶段清淤工作。加快城市绿化美化工程和山体公园建设，新增城市绿地700公顷，完成生态风景林建设1.2万亩。

　　（四）进一步深化改革开放，经济特区活力增强

　　创新重大项目用地报批工作机制和重点项目前期工作管理方法。推行重大片区项目审批体制改革，下放审批权限，充分发挥区街积极性。探索建立政府投资项目后评价机制，完善政府投资监管体系。稳步推进国有企业和事业单位改革。深化行政审批制度改革，推行窗口统一受理和送达，大力推进电子政务和网上审批，基本实现网上年检年审。整合全市有线电视网络资源，初步建立适应数字化发展需要的广播电视运行机制。深化公交管理体制改革，提高企业经营效益和服务水平。

　　发挥口岸优势，及时调整和完善外经贸扶持政策，推行“属地申报、口岸验放”、“多点报关、口岸验放”通关模式，全面实施入境货物集中查验，推行检验检疫直通放行制度，外贸进出口稳定增长。招商引资工作取得新成效，首次突破年实际利用外资20亿美元。企业增资势头强劲，增资项目274个，合同外资增资额达12.4亿美元，占全年合同外资总量65.7%。协议内资451.1亿元，增长3.47%，实际利用内资279.1亿元，增长33.5%。东亚海岸带可持续发展地方政府网络组织（PNLG）在厦设立秘书处，新增希腊马拉松市为友好城市，国际友城增至14个。

　　（五）发挥前沿平台作用，对台交流合作不断拓展

　　紧紧把握两岸关系重大积极变化带来的新机遇，及时推出对台先行先试新举措。全年实际利用台资（含第三地）7.2亿美元，增长15.1%。厦门与台湾的先进制造业、现代服务业对接有效推进，正新轮胎、友达光电、宸鸿科技等企业增资强劲。厦门市商业银行成功引进台资背景的香港富邦银行等战略投资者，成为大陆引进台资银行入股投资第一例。台湾人寿与建发股份合资的君龙人寿正式营业，成为全省第一家保险法人机构。台湾统一证券在厦设立代表处。厦门港成为率先开通两岸直航的大陆港口之一。东渡、五通“小三通”码头投入运营，厦金航线旅客年吞吐量达到90.3万人次，增长31.2%，厦金航线成为两岸人员往来的黄金通道。厦金、厦澎以及赴台旅游取得新突破，大陆13省市居民赴台旅游经厦门“小三通”航线、在厦暂住一年以上人员在厦办证赴台旅游顺利启动。厦门成为大陆先行开放5个周末包机客运直航和21个常态化客运包机直航航点之一，成为两岸直接通邮的封发局。厦航成为大陆在台设立办事处的第一家航空公司。厦门与澎湖、台中实现历史上首次空中直航。台湾中部四县市县市长同访厦门。大陆规模最大的台资医院——厦门长庚医院正式开业。大嶝对台小额商品交易市场改扩建工程启动。开展厦金航线海上搜救演习。厦金航线传染病联防联控和卫生应急机制实现常态化运行。“台交会”、“文博会”、“旅博会”、“食博会”和“世界金门日”等活动成功举办，海峡两岸民间艺术节、图书交易会、中医药论坛、农业合作论坛、中小学校长论坛、海洋文化论坛、海沧保生慈济文化节等活动影响进一步扩大。

　　（六）持续加强“三农”工作，社会主义新农村建设稳步推进

　　坚持以农民增收为目标，扎实推进农业产业化和农村城市化，农民人均纯收入增幅连续4年超过10%。新增2家国家重点农业龙头企业、4家福建省品牌金奖农业企业，市级以上农业产业化龙头企业实现总产值158.6亿元，增长18.8%。老区山区建设发展工程、移民造福工程稳步推进，23个重点村的旧村改造新村建设成效明显。农村市政基础设施建设步伐加快，自来水、有线电视入户率大幅提高，农村“家园清洁行动”扎实推进，农村客运、道路硬化、环境整治和文明建设卓有成效。在全国率先推行被征地人员基本养老保险，参保人数达4.9万人。新型农村合作医疗转型为居民基本医疗保险，将门诊常见病、多发病纳入保障范围，农村居民参保率达97%。在全省率先把被征地农民、退养渔民的就业再就业纳入扶持范围，全年完成农村劳动力转移1.97万人。开展农产品质量安全专项整治行动，建立健全农产品质量安全监管体系。

　　（七）弘扬一方有难八方支援精神，援川赈灾和对口支援工作取得成效

　　“5·12”四川汶川特大地震发生后，我市紧急向灾区派遣15支共767人的救援队伍，募集各类捐款3.34亿元、捐赠物资总价值2700多万元。提前完成1.7万套过渡安置房生产建设任务。圆满完成162名转运来厦伤病员的救治任务。接收了267名灾区学生到我市中职学校就读。迅速开展对口支援四川省彭州市龙门山等四个镇的灾后重建工作，开工建设10个项目。协助彭州市完成19个项目总投资35亿元的招商任务。与此同时，对口支援新疆、西藏、重庆、宁夏工作取得成效，厦漳泉龙城市联盟、闽西南五市和闽粤赣十三市区域合作进一步推进。

　　（八）推进“四个加强”、“四个破解”，和谐社会建设水平进一步提高

　　全市财政用于社会事业投资53.2亿元，增长29.8%。从2008年起连续三年，进行新一轮农村义务教育和公共卫生体系建设。完成20个中小学新建扩建项目，新开办学校16所，增加学位2万个。普通高中学生有95%在三级以上优质达标学校就学，进城务工人员子女有64%在公办中小学就学。五缘实验学校、厦门工商旅游学校、华厦职业学院、软件职业技术学院入驻新校区，集美大学新校区、华侨大学厦门校区、青少年综合实践基地、第二特教学校等项目顺利推进。顺利接收福建化工学校、集美轻工学校。厦门理工学院办学实力和社会影响快速提升。妇幼保健院二期扩建工程竣工，第一医院门急诊综合楼、中山医院内科综合病房楼、第三医院二期扩建工程建设进展顺利，厦门174医院获批成为三级甲等综合性医院。实施“医疗重组计划”，完成15家社区医疗服务中心新建、扩建工程以及30个标准化村卫生所建设。初步建立全国一流的医疗急救体系，有力防控手口足病、霍乱疫情发生，有序开展食用含“三聚氰胺”奶粉婴幼儿筛查治疗。加强人口计生工作，出生人口政策符合率95%，低生育水平保持稳定。国家乒乓球训练基地、工人体育馆等一批文化体育设施投入使用，厦门经济特区纪念馆、嘉庚纪念馆开馆，闽南戏曲艺术剧院、翔安体育中心等项目顺利推进。组建厦门社会科学院。制定文化产业发展和闽南文化生态保护区规划，扶持文化产业发展，中秋博饼、闽南童谣等项目入选第二批国家级非物质文化遗产保护名录。成功举办奥运火炬传递活动、第五届全国钢琴比赛、首届群众性体育联赛等活动。厦门国际马拉松赛再度成为国际田联路跑金牌赛事。

　　文明城市创建持续深化，以总分第一名通过“全国文明城市”复查考评。举办双高人才交流大会及海外留学人才与项目对接洽谈会，出台人才住房管理办法，全年引进接收各类人才2.8万人。强化职业技能培训和创业培训，全年新增就业21万人。率先建立广覆盖、多层次、可转移的城乡居民医保体系，被誉为“厦门模式”在全国推广。首创“建筑工地农民工大病保险”，社会保险覆盖面进一步扩大。连续三年提高企业退休人员基本养老金。对城乡生活困难群众实行临时物价补贴。社区居家养老服务工作取得新进展。社会保障性住房建设加快，已有2200个家庭选到满意的住房。加大中心城区交通环境综合整治力度，打通兴山路、天湖路等断头路，拓改湖明路、长岸路等交通拥堵点，交通改善工作取得成效。严格落实安全生产责任制，产品质量和食品药品安全专项整治取得明显成效。建立突发公共事件应急管理体系，加强防空防灾一体化建设，应急保障能力不断提高。扎实推进“平安厦门”建设，依法严厉打击各种刑事犯罪。重视群众来信来访，充分发挥市长专线电话功能，积极推进多元化纠纷解决机制建设，及时发现和化解不稳定因素。顺利完成十件为民办实事项目。外事、侨务、气象、防震、方志、档案、信息安全等工作得到加强。双拥共建、人防海防、民兵预备役、民族宗教、妇女儿童、老龄、残疾人等工作取得新成绩。

　　（九）加强民主法制建设，政府自身建设不断加强

　　全面推进依法行政，全年提请市人大常委会审议法规草案4项，制定和修订政府规章5项，扩大规范行政自由裁量权试点范围，建立市政府部门规范性文件前置法律审查制度。修订《政府工作规则》，完善行政决策机制。认真实施《政府信息公开条例》，依法公开政府信息，政务督查和责任追究制度不断健全。力倡勤俭节约，在压缩市、区行政事业单位公用经费5%支援灾区的基础上，再压缩市直各部门项目支出5%，保证民生支出和重点建设项目资金需求。认真贯彻《监督法》，认真执行市人大及其常委会的决议，坚持向人大及其常委会报告工作，自觉接受人大及其常委会法律监督和工作监督，认真办理人大代表议案和建议；支持人民政协履行政治协商、民主监督、参政议政职能，认真办理政协提案；全年办理人大代表议案4件、建议205件，政协提案501件。加强同民主党派、工商联、无党派人士、人民团体和社会各界人士的联系，认真听取他们的意见。落实领导干部廉洁自律规定，治理商业贿赂取得成效。聘请行风评议代表和特邀监察员，加强审计监察纠风工作，政府绩效评估再次位居全省第一。

　　各位代表！

　　刚刚过去的一年是极不平凡的一年，我们共同见证北京奥运会、残奥会、神舟七号载人航天飞行的圆满成功，共同经受重大自然灾害的严峻考验，共同应对国际金融危机带来的巨大影响。全市上下深入学习实践科学发展观，继续发挥经济特区先行先试作用，持续了“两个先行区”建设的良好态势和趋势。这是在中共中央、国务院和中共福建省委、省政府及中共厦门市委的正确领导下，在各级人大及各位代表的依法监督和有力支持下，在各级政协及各位委员的民主监督和积极参与下，全市干部群众齐心协力、艰苦奋斗的结果。在此，我谨代表市人民政府，向全市人民，向各民主党派、工商联和无党派人士、各人民团体、各界爱国人士，向离退休的老同志，向中央和省驻厦单位，向驻厦人民解放军、武警官兵和公安干警，向来厦的所有投资者和外来建设者，向所有关心支持厦门的港澳同胞、台湾同胞、海外侨胞和国际友好人士，表示衷心的感谢和崇高的敬意！

　　各位代表！我们也清醒地看到，我市经济社会发展还面临一些亟待解决的困难和问题，突出表现在：一是受国际金融危机影响，地区生产总值、工业、固定资产投资等部分经济指标增速回落，没有完成预期目标，经济增长速度减缓；二是发展具备竞争力的产业集群还需要进一步加强，产业链有待进一步延伸；三是金融危机引发的企业停产停业、减产减员、拖欠工资、债务纠纷等社会不稳定因素增多；四是征地拆迁难度仍然较大，一些重点工程的推进受到影响；五是国有资产监管措施需要进一步加强；六是城市管理、社会管理中一些群众关心的热点难点问题还没有得到有效解决，资源和环境压力较大，改善空气质量和节能减排的任务较为艰巨；七是一些公务员的服务意识和办事效率还有待进一步提高，有些工作还不够落实。对这些问题，我们高度重视，认真对待，在今后工作中将切实加以解决。

　　二、2009年的工作意见

　　2009年，是新中国成立60周年，也是推进“十一五”规划顺利实施的关键一年。当前国际金融危机正在从局部发展到全球，从发达国家传导到新兴市场国家和发展中国家，从虚拟经济扩散到实体经济，波及范围之广、影响程度之深、冲击强度之大超出人们预料。经济社会发展中一些矛盾可能更加突出，各种不利因素对我市的叠加影响不可低估。面对复杂多变的宏观发展环境，我们要始终保持清醒头脑，增强忧患意识，把克服金融危机的影响转化为优胜劣汰、转型升级、转变职能的契机，充分依靠我市日益增强的综合实力和抵御风险能力，充分利用我市新一轮跨越式发展集聚的坚实基础和发展后劲，充分发挥扩大内需促进经济增长和国家支持海峡西岸的政策作用，不断拓展经济特区先行先试的效应，化挑战为机遇，化压力为动力，坚定战胜困难的信心，持续推进经济社会平稳较快发展。

　　政府工作的总体要求是：全面贯彻党的十七大和十七届三中全会精神，高举中国特色社会主义伟大旗帜，以邓小平理论和“三个代表”重要思想为指导，认真学习实践科学发展观，全面落实省委关于在海峡西岸建设“两个先行区”的重大部署，把“四求先行”、“四个重在”落实到政府工作的各个方面，千方百计保增长、扩内需、调结构、保民生、保稳定，把扩大内需作为保增长的根本途径，把加快转型升级作为保增长的主攻方向，把深化改革扩大开放作为保增长的强大动力，把改善民生作为保增长的出发点和落脚点，坚持项目带动、品牌带动、创新带动和服务带动，大力开拓国内国际两个市场，继续推进“四个加强”、“四个破解”，促进经济社会又好又快发展，努力在海峡西岸建设科学发展的先行区、两岸人民交流合作的先行区中作示范当榜样。

　　2009年主要预期目标建议为：地区生产总值增长11%左右；规模以上工业总产值增长11%；全社会固定资产投资增长10%；外贸出口增长12%；实际利用外资15亿美元；地方级财政收入增长11%左右；社会消费品零售总额增长16%；城镇居民人均可支配收入和农民人均纯收入分别增长9%和8%；居民消费价格指数104左右；城镇登记失业率控制在4.5%以内；人口自然增长率控制在9.5‰以内。单位生产总值能耗降低2.52%，二氧化硫排放量和化学需氧量减排提前一年达到“十一五”目标。在实际工作中要加倍努力，同时，要根据形势的变化，进行适当调整。

　　实现上述目标，我们必须扎实做好以下工作：

　　（一）力促企业健康运行，确保经济稳定增长

　　全力以赴帮助企业渡过难关。保持经济的增长，首要的任务就是要保企业。要以保产值、保利润、保就业为目标，进一步加强和改善企业服务。一是分门别类研究企业生产经营情况，建立受理反映问题的服务平台，“一企一策”扶持企业。对全市工业企业重新梳理，新确定一批重点帮扶企业。二是推动金融机构对企业的支持。鼓励银行等金融机构对基本面比较好、有市场、有订单但暂时出现经营或财务困难的企业给予信贷支持，切实帮助企业解决融资困难。建立转型升级重点项目贷款贴息风险保证金制度，引导创业投资基金规范运作。三是帮助企业开拓市场。建立地产名优产品推荐目录，引导商贸业、建筑业与工业企业相互配合，在政府采购、重点建设和市政工程项目中，采取签订中长期直接供货合同方式，加大地产品购销力度。注重发挥厦门出口商品展销会等平台作用，鼓励本地企业开展电子商务，大力拓展市场。四是减轻企业负担。限时取消和停止不符合规定的收费，进一步规范行政事业性收费，规范中介机构、公共服务行业、社会团体和行业协会的收费行为。五是减征部分涉企收费。在保持职工原有待遇不变的情况下，本年度对企业工伤保险金、生育保险金、失业保险金、残疾人就业保障金、人事代理服务费减半征收，对基本医疗保险费单位缴交部分的费率下调一个百分点。包括其他涉企收费项目的取消和减免，以及先前出台的房产政策、出口扶持政策等，让利企业和个人达12亿元。

　　保持出口稳定增长。一是推进出口市场多元化，在稳定北美、欧洲、日本等传统市场的基础上，积极开拓中东、俄罗斯、拉美、非洲等新兴市场。二是帮助出口加工和贸易企业充分利用国家提高出口退税和取消、降低出口关税等有利政策，积极开拓国际市场。健全银贸协作机制，帮助外贸企业解决资金困难。三是鼓励企业开拓出口市场，更多地采用出口信用保险和保单融资，进一步降低出口风险。四是对我市大宗出口商品的一般贸易予以支持，稳定大宗商品出口。五是大力在内陆地区开辟“无水港”，积极拓展海铁联运，增加港口吞吐量和进出口额。六是改善软硬件条件，提高通关效率，吸引在厦口岸出口的外地企业来厦门设立生产经营机构，吸引更多的省内外货物从厦门港进出，大力促进中转业务。七是办好第四届中国（厦门）品牌产品国际采购交易会等展会，有针对性地组织企业到境外参展，鼓励企业建设自主营销的境外终端贸易网点，扩大厦门品牌产品的知名度和影响力。八是扶持进出口行业协会发展，有效应对国际贸易壁垒。九是把鼓励进口与促进企业设备更新结合起来，扶持企业进口先进的技术设备。十是培植优势出口产业基地。继续完善信息技术、光电产品、新材料、汽车等国家级和船舶、服务外包等省级出口基地建设，推进体育用品、医疗器械、水暖器材、工程机械、油画等商品出口基地建设。

　　（二）进一步加大投资力度，千方百计扩大内需

　　力促投资较快增长。全年安排重点建设项目投资448亿元，主要投向产业发展、基础设施、民生保障、生态环境建设等领域，拉动民间资本进入符合国家产业政策导向的行业和领域。一是注重工业集中区等重点片区的投入，加快新站营运中心、杏林湾营运中心建设，完善重点片区的金融、商贸、电讯、公交、学校、医院、供水供电、文化娱乐等配套建设，推进110千伏及以上输变电工程、东部燃气电厂等项目建设进度。二是注重新的经济增长点、财税点、就业点的投入，推动景智光电、新科宇航、建颖科技等一批项目启动建设，加快推进金桥卷烟生产线技改、厦顺铝箔、麦克奥迪、钨业新能源等一批项目，促进玉柴发动机、金龙车身二期以及友达光电三期等项目建成投产。三是注重重大基础设施项目的投入，力争翔安隧道建成通车，成功大道、海翔大道实现全线通车，福厦铁路（厦门段）、厦门新站投入使用，加快推进龙厦铁路、厦深铁路、厦门至安溪高速公路、沈海高速公路（厦门段）扩建工程、厦漳跨海大桥建设，开工建设厦成高速公路（厦门段）、前场铁路大型货场。力争海沧港区14＃－19＃泊位、东渡港区现代码头工程等10个深水泊位基本建成并投入使用，继续推进海沧航道扩建二期、机场三期扩建工程等项目建设。四是注重民生保障项目的投入，加大旧村、旧街、旧片区改造，大力推进湖边水库、曾厝垵等片区综合改造，适时启动西郭、枋湖等一批片区的旧城改造。加快建设北溪引水左干渠改造二期工程和东部固废中心。续建快速公交系统。五是注重策划生成储备一批重大项目，积极做好长泰枋洋水利枢纽工程、莲花水库、第二东西通道等项目的前期工作。六是注重为项目建设创造有利条件，在保持征地拆迁总体政策不变的前提下，有效推进征地拆迁工作。

　　努力扩大消费。一要切实提高消费能力。把扩大消费与完善收入分配政策相结合，与扩大就业相结合，千方百计增加城乡居民收入。继续提高企业退休人员基本养老金。二要积极引导住房消费。落实购买普通商品住宅的各项优惠政策，研究出台购买岛内重点片区商品房的落户政策，制定满足居民购买改善型住房需求的办法，培育发展二手房市场和房屋租赁市场。引导房地产企业优化供应结构，进一步完善房地产市场信息披露制度，健全房地产市场预警预报体系，切实保障房地产消费者合法权益。三要鼓励旅游消费。在发展传统观光旅游消费的基础上，加快发展邮轮、游艇、帆船及其他高端旅游产品，推进重点旅游项目建设，争取举办更多有影响力的展会，提升厦门旅游吸引力。积极发展森林旅游、温泉旅游、休闲渔业等项目，支持建设乡村休闲度假旅游区，鼓励发展“农家乐”。四要发展汽车消费。落实居民购买节能环保小排量汽车的优惠政策，鼓励金融机构发展汽车消费信贷业务，加强停车场建设和管理，并研究居民汽车消费的鼓励措施，促进消费升级。五要推进服务消费。继续推进闽南文化生态保护实验区和闽南戏曲艺术剧院、闽台戏曲大观园等文化设施建设，培育闽南特色的文化产业。积极发展商品油画、钢琴、艺术品、影视动画、广告策划等文化创意产业。抓好特色商业街、高档购物商场建设，扩大年货展、礼品展等各类节庆消费。六要注重农村消费。鼓励农村商业设施建设，支持商贸企业向农村延伸经营网络，扩大消费品在农村的覆盖面。做好“家电下乡”推广工作。七要改善消费环境。认真落实产品质量安全责任制，进一步整顿和规范市场秩序，强化生产流通的全过程监管，严格市场准入，加强行业自律，确保食品药品的安全，让群众放心消费。

　　（三）提高自主创新能力，调整优化产业结构

　　推进产业集聚提升。充分发挥工业集中区等园区的载体作用，引导产业集聚，做强做大电子、机械、化工等支柱产业，加快发展食品、轻工等传统产业，重点扶持光电、生物医药、新材料、新能源等高新技术产业。推进液晶面板向上下游延伸，发展发动机、高压油缸、液压元器件等汽车和工程机械关键零部件项目，继续引进电工电气等优势产品项目，推动光伏产业与LED的结合，不断延伸产业链。培育壮大一批百亿元以上产值的企业和产业集群。鼓励企业利用国家增值税转型契机进行技术改造，全力支持高成长型的品牌骨干企业加快发展，促进产业升级。深入实施品牌带动战略，发挥好厦门现有品牌的作用，做好策划和宣传，在人才引进、政府采购等方面优先给予支持，着力引进一批国内外知名品牌企业。加强海洋规划、开发和保护，管好用好港口岸线，发展海洋运输、滨海旅游、海洋生物制药等海洋产业，办好“国际海洋周”，建设海洋经济强市。

　　大力推进自主创新。市财政投入科技创新、研发资金4.6亿元，用于完善和推动厦门工业产品设计中心、动漫产业基地数字媒体等公共技术服务平台建设，加快建设火炬东海科技城，新建一批工程（技术）研究中心和实验室。做好高新技术企业的认定，引导企业加大研发投入，继续出台有利于自主创新的扶持政策，推动企业提高自主创新能力。鼓励企业与科研院所合作，加速科技成果转化。实施标准化战略，启动政府质量奖，提高产品质量。加强知识产权创造、使用、管理和保护，积极创建“国家知识产权示范城市”。

　　加快发展现代服务业。围绕提高服务业增加值和就业比重，落实好已出台的服务业发展实施意见以及相关领域扶持政策，用好每年1亿元服务业发展引导资金，吸引社会资本投向现代服务业，重点加快发展电子商务、现代物流、金融保险、会展等生产性服务业，加快发展旅游、商贸等生活性服务业。实现海沧保税港区一期封关试运行，确保火炬（翔安）产业区保税物流中心（B型）投入运营，加快推动象屿保税区、保税物流园区和东渡港区的功能整合、政策叠加。支持金融业发展，鼓励金融产品创新，推动企业股份制改革，支持上市公司再融资，鼓励上市公司并购重组，支持有条件的企业发行企业债券。改善软硬件环境，办好“国际动漫节”，培养壮大软件和信息服务业。完善“无线城市”建设，促进移动互联网产业发展。推进观音山、五缘湾、杏林湾、环东海域等商务营运中心建设，积极吸引国内外企业在厦设立地区总部、销售中心、结算中心和研发中心，争取更多的国内外中介机构落户厦门。

　　大力实施人才强市战略。坚持培养和引进并举，继续办好各类培训和引才引智活动，拓宽人才资源开发与交流合作渠道，着力培养引进一批社会急需紧缺的各类人才，优化我市人才结构。开展校企合作、订单培训，实施重点企业优秀青年技术工人技能培训，培养我市产业发展所需的技能人才。完善人才服务体系，健全激励机制，真心实意关心人才、爱护人才、凝聚人才，营造尊重知识、尊重人才、尊重创造的良好环境，为经济社会发展提供人才保障。

　　（四）坚持经济特区先行先试，更好发挥示范榜样作用

　　深化体制机制创新。落实好国家和省里统一部署的重大改革措施。深化港口一体化改革，推进厦门湾南北两岸“同港同政策”的落实。整合优化市属国有企业，加强风险防范管理，促进国企做强做大和国有资产保值增值。推进投融资体制改革，积极培育资本市场。深化行政管理体制改革，做好政府机构改革。加强电子政务建设，推进审批制度改革。大力推进公共资源市场化配置改革，推行全市机关事业单位大宗货物政府采购，加强土地招拍挂、建设工程招投标、药品和医疗器械招投标等市场建设，完善全市统一的公共资源交易平台。全面推行公务卡。稳步推进教育、文化、卫生等社会领域改革，增强社会事业发展的活力。

　　大力做好招商引资。围绕促进产业结构优化升级，围绕提高自主创新能力，围绕增强中心城市服务功能，更加突出招商重点，创新招商方式。继续办好“投洽会”、“台交会”、“石材展”等重大展会活动，提升厦门对外开放的良好形象。加强与国内外著名投资咨询中介机构的联系与合作。继续做好工业集中区、商务营运中心和其他重大片区的项目引进工作，鼓励现有企业增资扩产。加快服务外包基地建设，引进和培育一批服务外包骨干企业。以发展光电、软件等新兴产业集群为主要目标，以火炬（翔安）产业区、软件园产业基地等园区为载体，对接国际资本转移。进一步扩大对内开放，吸引国内企业来厦设立各类生产经营机构。落实好对民营企业融资担保、贴息等扶持政策，大力发展中小企业和民营经济。

　　促进两岸产业对接。进一步办好海沧、杏林、集美三个国家级台商投资区，增强对接台湾先进制造业和现代服务业转移的载体功能，吸引更多的台湾企业和上市公司落户厦门，引进台资企业的大陆营运总部，促进台资企业增资扩产和以商招商，提升两岸经贸合作层次。加强与台湾的科技合作，争取成为“对台科技合作与交流基地”。继续做好与台湾的金融合作，积极引进台湾金融机构来厦投资设立证券、保险、基金等公司，争取成为“海峡两岸金融合作试验区”。办好厦门台湾水果销售集散中心、大嶝对台小额商品交易市场。

　　进一步扩大“三通”效应。积极做好来厦暂住人员“厦金游”，打造厦门旅游新亮点。加大厦金航线宣传推介，改善客运航线和接待条件，扩建五通厦金海空联运码头，增加运营船舶和航班数量，吸引大陆二十五省（区）市居民经“小三通”或厦门直航点赴金门、澎湖和台湾本岛旅游。做好空中、海上客货运直航，推进空中直航截弯取直和航线优化，力争实现两岸常态包机腹舱载货，争取开通海峡客货滚装运输业务。发展对台邮轮经济，争取开辟厦门至台中等港口的海上客运航线和厦门—澎湖—台湾本岛邮轮客运航线。做好厦台两岸邮件封发局工作。建立和完善海峡两岸搜救、防污应急机制。推动铺设与金门海底通信电缆，争取对台通信先行一步。

　　拓展对台文化交流。充分利用闽南文化的特色和优势，深化与台湾的教育、文化、卫生、体育、民俗、宗教等方面的交流交往。办好首届“海峡论坛”。推进海峡两岸中医药交流合作中心建设，争取在市中医院设立台胞中医门诊部。做好在厦台湾地区人才专业技术职称评审试点工作。继续办好海峡两岸“文博会”、龙舟赛、民间艺术节、图书交易会、保生慈济文化节等重大节会活动。继续发挥好厦门卫视、闽南之声广播、厦门网等媒体作用。

　　（五）推进社会主义新农村建设，千方百计促进农民增收

　　继续提高农民生活水平。深入贯彻十七届三中全会精神，努力破解各种发展难题和体制机制障碍，促进农民持续增收。继续开发公益性和服务性岗位，鼓励工业集中区、新城区用地单位和企业优先吸纳被征地农民、退养渔民就业，优先支持他们参与工业集中区、开发区的配套服务，鼓励他们以创业带就业，力争全年转移农村劳动力1.5万人。适当降低被征地农民基本养老保险参保门槛，实行被征地人员老年补助办法，扩大农村社保覆盖面。完善农村最低生活保障制度，适当提高保障标准。改善农村文化设施，完成“农家书屋”工程建设，丰富农民文化生活。

　　加快农村城市化。继续推进城乡统筹发展，通过工业集中区、重大片区、重大产业项目带动农村城市化。做好“镇改街、村改居”后续工作，加快推进农村社区建设，扩大农村公共服务范围。继续加大农村道路交通、供水、通信、垃圾处理等基础设施建设，加快边远山区饮水安全工程建设，提高农村客运通行政村覆盖率、有线电视入户率。推进新一轮农村义务教育和公共卫生体系建设。继续推动20个行政村实施旧村改造新村建设，新启动12个老区山区村改造建设，稳步推进边远山区和水源保护区的移民造福工程，探索建立重要水源保护区生态补偿机制。继续抓好和完善“金包银”、“金包金”项目，加大“金边”招商招租和股份配售力度，鼓励成立规范的股份公司进行经营管理。完善农村宅基地制度，做好农村房屋登记发证工作。

　　推进农业产业化。完善农村土地承包经营权管理体制，探索建立规范的土地承包经营权流转机制。实施“两头在厦、中间在外”农业发展战略，大力开发优势种苗业、高附加值农产品加工业。扶持建立农民专业合作社，再建一批无公害农产品生产基地。加大对农业产业化龙头企业的扶持力度，拓展订单农业，推进“一村一品”、“一村一业”特色农业及生态休闲农业发展。加强土地整理工作和水利设施建设。加强农产品质量安全监管工作，强化重大动植物疫病防控。

　　（六）加强城市生态环境建设，持续提升城市品位

　　统筹推进岛内外发展。做好土地利用总体规划修编工作，完善城市规划体系和城市规划实施机制。加大规划设计投入，主动适应城市经济社会发展的变化，对一些规划不到位、不完善、不细致的地方进行完善。加快集美、海沧、同安、翔安新城区建设，大力推进环东海域、五缘湾、杏林湾、湖边水库、厦门新站等重大片区开发建设，并以此带动其他区域全面发展。充分发挥各区各自特色，找准定位，努力避免功能和产业的同质化，实现差异化发展。

　　提高城市管理水平。整合信息资源，完善城市基础地理信息数据和管理信息共享平台，加快推进城市交通信息公共平台建设。继续推进统一的数字城管系统建设，实现城管信息化、网络化，全面提高城管执法能力和管理水平。加大城市公共设施建设力度，扩建石胄头和同安污水厂，加快完善污水处理及垃圾综合利用、无害化处理体系，加强城市建筑和市政设施的后续管理和综合整治。完善查处违法建设的配套政策，加大力度处理历史遗留违法用地、违法建筑问题，有效遏制违法建设。

　　推进生态文明建设。认真落实环保目标责任制，做好新一轮国家环保模范城市创建工作。加强水源保护与治理，推进环东海域、筼筜湖、五缘湾、杏林湾水库、九龙江流域和集美深青溪等综合整治，全面启动厦门海域清淤整治工程，修复沙滩、红树林等典型海洋生态系统，加强中华白海豚等珍稀海洋物种的保护。推进滨海岸线生态风景林建设，加快天竺山等森林公园、下潭尾等湿地公园和火烧屿白海豚救助繁育中心等项目建设。实施宁静工程，继续推进岛外林相改造和市区垂直绿化工程，加快全国环境优美镇、绿色社区、绿色学校、生态村建设。继续抓好节能减排，深入实施循环经济示范工程，全面落实投资项目节能评估和审查制度，鼓励发展精装商品房，积极推广使用天然气、水煤浆等清洁能源，扩大污水综合回用，强化机动车排气污染控制，加强扬尘污染控制，改善空气质量。

　　（七）坚持统筹协调，全面推进社会事业发展

　　加快发展教育事业。促进城乡义务教育均衡发展，推动优质教育资源向农村辐射。全面推进素质教育，深入实施课程改革，减轻中小学生过重课业负担。加快中小学义务教育项目建设，推进新建工业集中区和新城区配套学校项目建设。继续共建厦大、集大，推进厦门理工学院二期、华侨大学在建工程建设，开工建设厦大翔安新校区。实施中小学校舍抗震安全加固改造工程。加强教师队伍建设，切实提高教师特别是农村中小学教师素质和待遇。扶持和规范民办教育，加强学前教育、职业教育、社区教育和继续教育，大力发展华文教育，扩大教育国际合作与交流。

　　提高医疗服务水平。完善社区医疗服务体系，推进社区医疗服务中心的标准化和农村卫生院建设。做好中医院、第二医院、翔安医院等项目建设，开展福建（厦门）－新加坡友好医疗服务中心前期工作。逐步扩大医疗救助和医疗惠民范围，推广市民健康信息系统，加强医德医风建设，提高医疗服务质量。普及医疗急救知识，不断完善公共卫生应急体系。加强人口计生工作，稳定低生育水平，综合治理出生人口性别比偏高问题，提高出生人口素质。

　　推进文化体育事业繁荣发展。继续推进小白鹭艺术中心以及同安文化中心等一批区级文化基础设施项目建设。继续做好第三次全国文物普查，做好鼓浪屿申报世界文化遗产前期工作。发挥厦门社会科学院作用，繁荣发展哲学社会科学，积极发展新闻出版、广播影视、文学艺术事业，加强档案保护，续修《厦门市志》。加强科普工作，不断提高市民科学素养。推进公共文体服务项目建设，着力丰富农村、偏远地区居民和农民工的精神文化和体育生活。办好第十一届中国戏剧节、全国文化院校奖钢琴比赛等赛事，推进“厦门世界音乐节”前期筹备工作。推动翔安体育中心、体育运动学校、环岛路水上帆船帆板基地和杏林湾皮划赛艇基地建设，继续办好厦门国际马拉松赛等活动，做好第十一届全运会参赛工作。

　　加强对外交流和区域协作。按照国际化的城市定位，创新交流合作方式和内涵。加强友好城市、友好港口之间的经贸文化交流合作。加快领馆区建设步伐，争取新设外国领事馆。坚持服务周边、服务海西、服务全国，进一步加强厦漳泉龙城市联盟，密切闽西南五市、闽粤赣十三市的产业、旅游等实质性合作。认真实施援建彭州地震灾区灾后重建项目，继续开展支医、支教和就业援助。做好西藏、新疆、宁夏等对口支援工作，实现共同发展。

　　（八）注重民生保障，提高和谐社会建设水平

　　突出抓好就业和社会保障。加快完善覆盖城乡的就业服务体系，重视大中专毕业生和新增劳动力就业，加强岗前技能培训，建立人力资源供求信息分析评估制度和企业关闭停产报告制度。实施积极就业政策，大力发展各类社区服务业，不断增加就业容量。完善面向困难群众的就业援助制度，巩固消除“零就业”家庭工作成果。继续做好农民工社会保险工作，基本实现建筑施工企业农民工参加工伤保险。建立健全全市统一的城乡一体化基本医疗保险制度，覆盖城镇居民、农村居民、未成年人和大中专院校在校生。由政府出资，为全市市民办理自然灾害公众责任险，在台风等自然灾害造成伤亡时提供救助保障。加强社会保险基金监管，确保基金安全。继续实行和完善对低收入群体的动态补助机制，确保城乡困难家庭、离退休职工、在校贫困大学生基本生活水平不下降。

　　持续办好惠民实事。继续推进社会保障性住房建设，保质保量完成已开工项目，做好分配管理工作。继续推动交通改善，大力发展城市公共交通，抓好普通公交、快速公交、出租车和农村客运的运营管理工作，提升客运服务质量。建设岛内货运专用通道。新建保险事故车辆拆检定损中心，进一步完善交通事故快速处理办法。完成市级粮食储备库建设，提升粮食安全保障能力。探索建立重要副食品生产和供应的保障机制，加强价格监测监管，保持价格总水平基本稳定。加强应急避难场所建设，精心组织市民参与防空防灾应急疏散演练，提高市民应急避难和自救互救能力。完善社会救助体系，发展公益事业和慈善事业。重视残疾人事业发展，建设残疾人庇护工场。加强老龄工作，推动社会化养老服务体系建设。发挥工会、共青团、妇联等群团组织以及行业协会作用。做好民族与宗教工作，加强国防教育和拥军优抚安置，争创全国双拥模范城“七连冠”。

　　切实维护社会安全稳定。积极深化“平安厦门”建设，加强社会治安综合治理，健全完善治安防控机制，强化“城中村”整治，切实加强路面防控，依法严厉打击各类违法犯罪活动，保障人民生命财产安全。落实安全生产“一岗双责”和行政问责制，继续抓好海陆交通、危险化学品、非煤矿山、森林火灾、建筑施工等安全专项整治，切实加强隧道安全管理，遏制重特大事故发生。做好群众来信来访、法律服务、法律援助和人民调解工作，完善多元化纠纷解决平台建设和工作机制，发挥好市长专线电话功能，建立健全领导接访长效机制，及时化解各类社会矛盾。强化劳动监察，提高劳动争议仲裁效能，构建和谐劳动关系。完善重大危险源监控、应急管理体制机制和预案体系，加强气象防灾减灾和应对气候变化能力建设，提高保障公共安全和处置突发事件能力。

　　巩固提高全国文明城市创建成果。正确把握人民群众对文明城市建设的新要求新期望和文明城市创建工作的新特点新问题，不断完善运作机制，持续提高创建工作水平。大力推进社区建设，完善社区事务“一门式”服务工作机制，拓展与群众生活密切相关的服务内容，优化办事流程，方便群众办事。重视居委会、村委会规范化建设，加强对基层组织换届选举的指导和监督。提升社区物业服务水平。促进各类社会组织规范有序发展，推进社会工作人才队伍建设，积极倡导志愿者服务，引导和鼓励市民参与社会公益活动，共建共享文明温馨家园。

　　三、加强政府自身建设，不断提高行政能力和服务水平

　　在依法行政中体现效能。健全重大事项专家咨询、公众参与、集体决策制度。加强政府立法工作，不断提高立法质量。全面推行规范行政自由裁量权工作，开展行政机关规范性文件清理，加强行政复议和行政应诉工作。继续开展民主评议政风行风活动，全面实施政府绩效评估，完善决策、执行和考核监督体系，健全岗位责任制和重点工作责任制，强化行政问责和政务督查。健全政府职责体系，加强条块之间的沟通合作，密切部门之间的协调配合。自觉接受市人大及其常委会的监督，自觉接受市政协的民主监督，坚持定期向市人大及其常委会报告工作和向市政协通报情况。认真听取民主党派、工商联、无党派人士和各人民团体的意见。认真做好人大代表议案、建议和政协提案的办理工作，重视新闻舆论和社会公众监督，支持监察、审计部门依法独立监督。依法深入推进政府信息公开，完善政府公报、政府网站、政府新闻发布会、政府信息查阅场所等公开渠道，保障人民的知情权、参与权、表达权、监督权。

　　在加强服务中体现责任。越是在经济发展遇到困难的时候，越要做好政府服务。要带着感情，带着历史的责任感去服务社会、服务企业、服务群众。要根据变化的形势，主动调整服务方向、服务方式，把服务重点放在培育和壮大新的经济增长点、财税点、就业点，放在转变发展方式、调整优化结构，放在增加市民收入，为企业、为基层、为群众多办实事。要深入基层，深入企业，有针对性地帮助企业解决各类问题。发挥流动人口管理站的作用，为外来员工提供优质高效服务。政府公务员要加强法律和政策的学习领会，加强对新领域、新课题的研究探索，提升素质和能力。做好第二次经济普查，摸清产业结构变化情况，为全市经济社会发展提供确实可靠依据。启动“十二五”规划前期工作。

　　在先行先试中体现作为。敢闯敢试、先行先试，是经济特区发展的灵魂。认真履行经济特区使命，继续推进改革开放和制度创新，大胆探索，勇于实践，创造性地用活用足各项优惠政策。加快建立健全经济社会发展综合评价、科技创新、产业发展、城市规划、土地供给、环保约束、财税分配、民生保障、舆论引导、干部实绩考核等方面的体制机制，为科学发展提供制度保障。

　　在廉洁从政中体现作风。认真贯彻落实中纪委十七届三次全会精神和省纪委八届五次全会精神，更加注重治本，更加注重预防，更加注重制度建设，认真落实“一岗双责”。全面推进“阳光工程”，严格规范规划、用地、工程招投标、财政资金使用、政府采购、国有资产转让、工程质量监理等公共资源管理权力的运行，继续开展治理商业贿赂，杜绝损害群众利益的不正之风。加强对扩大内需促进经济发展政策措施执行情况的检查，保证政策措施落实到位。增收节支，厉行节约，在2008年实际预算安排的基础上，继续按5%的比例压缩公用经费和业务经费，把有限的资金用在最能产生经济和社会效益的项目上。严格控制公务消费，削减一般性开支，在车辆、会议、公务接待、出国（境）等方面费用保持零增长，努力降低行政成本，始终保持艰苦奋斗的光荣传统。

　　各位代表！让我们更加紧密地团结在以胡锦涛同志为总书记的党中央周围，高举中国特色社会主义伟大旗帜，以邓小平理论和“三个代表”重要思想为指导，深入学习实践科学发展观，加快构建社会主义和谐社会，在中共厦门市委的坚强领导下，坚定信心，同心协力，攻坚克难，开拓创新，为推进海峡西岸“两个先行区”建设作出新的贡献，以优异的成绩迎接新中国成立60周年！
